# Phenome-wide association study of monogenic inflammatory bowel disease genes in diverse biobanks identifies population-specific and shared Goldilocks alleles: implications for Precision Medicine

**DOI:** 10.1093/ecco-jcc/jjaf098

**Published:** 2025-08-05

**Authors:** Michelle M Bao, Meltem Ece Kars, David Zhang, Kyle Gettler, Daniel Rader, Scott Snapper, Yuval Itan, Judy H Cho

**Affiliations:** Division of Pediatric Gastroenterology, Susan and Leonard Feinstein Inflammatory Bowel Disease Center, Icahn School of Medicine at Mount Sinai, New York, NY, United States; Department of Pathology, Molecular, and Cell Based Medicine, Icahn School of Medicine at Mount Sinai, New York, NY, United States; The Charles Bronfman Institute for Personalized Medicine, Icahn School of Medicine at Mount Sinai, New York, NY, United States; Department of Genetics and Genomic Sciences, Icahn School of Medicine at Mount Sinai, New York, NY 10029, United States; Mindich Child Health and Development Institute, Icahn School of Medicine at Mount Sinai, New York, NY 10029, United States; Department of Genetics, Perelman School of Medicine, University of Pennsylvania, Philadelphia, PA, United States; Department of Pathology, Molecular, and Cell Based Medicine, Icahn School of Medicine at Mount Sinai, New York, NY, United States; Department of Genetics, Perelman School of Medicine, University of Pennsylvania, Philadelphia, PA, United States; Division of Gastroenterology and Nutrition, Boston Children’s Hospital, Boston, MA, United States; The Charles Bronfman Institute for Personalized Medicine, Icahn School of Medicine at Mount Sinai, New York, NY, United States; Department of Genetics and Genomic Sciences, Icahn School of Medicine at Mount Sinai, New York, NY 10029, United States; Mindich Child Health and Development Institute, Icahn School of Medicine at Mount Sinai, New York, NY 10029, United States; Department of Pathology, Molecular, and Cell Based Medicine, Icahn School of Medicine at Mount Sinai, New York, NY, United States

**Keywords:** Monogenic inflammatory bowel disease, PheWAS, NPC1, ADA, ADA2

## Abstract

**Background and Aims:**

Monogenic forms of inflammatory bowel disease (IBD) are driven by variants in genes critical to pathways in intestinal homeostasis and immunity. We investigated gene- and variant-level effects of these genes with IBD and phenome-wide association, leveraging large-scale whole exome sequencing data across 4 diverse cohorts: BioMe Biobank (Regeneron and Sema4), Penn Med Biobank, and UK Biobank.

**Methods:**

Predicted loss- and gain-of function variants were extracted from 102 monogenic genes. Gene- and variant-level association tests for binary traits were performed across 4 cohorts grouped based on genetic similarity in European, African, and Admixed American populations.

**Results:**

From 11 546 variants extracted, over two-thirds were predicted as loss-of-function (LOF), with 93% classified as ultra-rare and 1172 Goldilocks variants (not ultra-rare) enriched at least 10-fold in African populations. Gene-level IBD association testing demonstrated numerous replicated associations in European cohorts, reflecting well-powered independent cohorts. Twenty monogenic genes overlap with genome-wide IBD loci, fifteen of which displayed gene-level association trends. Heterozygous carriage of African-predominant LOF alleles in *NPC1* (intracellular cholesterol transport) and *ADA*/*ADA2* (purine metabolism), were associated with IBD. These variants also showed replicated associations with phenotypes related to cardiac conduction, infection, and lipid metabolism.

**Conclusions:**

We define overlap between monogenic and genome-wide IBD loci and reveal population-specific allelic heterogeneity of IBD risk genes. We uncover novel phenotype associations suggesting pleiotropic effects of monogenic IBD genes. African-predominant variants revealed allelic associations absent in European cohorts, and of potential clinical significance, underscoring the importance of increasing diversity in genetic studies.

## Graphical Abstract

**Graphical Abstract:**
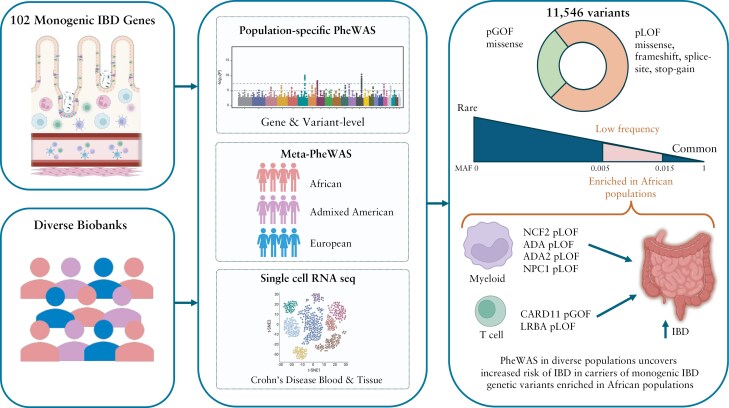

## 1. Introduction

Inflammatory bowel disease (IBD) is a group of disorders, encompassing Crohn’s disease (CD) and ulcerative colitis (UC), leading to chronic, immune-mediated inflammation of the gut. IBD arises from a complex interplay between host-microbe interactions, environmental factors, and genetic susceptibility. Genome-wide association studies (GWAS) have identified over 300 associated genetic loci in polygenic IBD, primarily derived from European (EUR) populations with 1 recent large-scale IBD GWAS reported in East Asians.^1,2^ IBD loci can display population-specific variation, with *NOD2* alleles being a notable example of conferring significant risk in EUR populations while being absent in individuals of African (AFR) ancestry.^3^ Despite recent efforts that have expanded to include non-EUR populations,^2,4,5^ they remain substantially underrepresented in genetic studies. With the incidence of IBD rising in non-EUR groups, further research is needed to understand the genetic architecture of IBD across diverse populations.^6,7^ Furthermore, as the ancestral population, AFR individuals carry multiple relatively uncommon, low-frequency alleles with major therapeutic and epidemiologic effects (ie, Goldilocks alleles), such as with loss-of-function (LOF) alleles in *PCSK9* conferring low LDL values.^8^

Monogenic IBD, driven by rare LOF or gain-of-function (GOF) variants, typically manifests as a very early onset disease, often accompanied by extraintestinal systemic manifestations.^9^ Monogenic IBD genes are critical to immune response, epithelial barrier maintenance, and microbial defense, potentially offering unique insights into mechanisms of intestinal inflammation. Rare variants are inherently more likely to be population-specific and may have implications for polygenic, later-onset IBD, especially in under-studied AFR cohorts. Polygenic risk scores (PRS) can be developed from common variant GWAS data,^10^ generating multi-SNP panels predicting several-fold increased risk in population-based data for top compared to lower risk groups. The addition of even very small AFR-American IBD case-control reference data improved the prediction of IBD by PRS in EUR ancestry and admixed (ie, LatinX) cohorts.^11^ Likely reflecting the greater genetic diversity and shorter linkage disequilibrium blocks (contiguous genetic regions with low recombination rates, carrying genetic variants typically inherited in tandem) present in AFR populations, PRS predictive enrichment in AFR IBD cases was poor; this undoubtedly reflects the small contributions of AFR IBD cases in present IBD GWAS reference datasets.

To generalize and prioritize these genes, we performed a comprehensive phenome-wide association study (PheWAS) of monogenic IBD genes, leveraging large-scale whole exome sequencing (WES) data from multiple, randomly ascertained biobanks in the United States, as well as the UK Biobank. We performed gene- and variant-level analyses across EUR, AFR, and Admixed American (AMR) populations, followed by population-specific meta-analyses. We demonstrate shared and unique association trends in AFR-predominant variants with IBD and other related phenotypes.

## 2. Materials and methods

### 2.1. Study cohorts and phenotypes

**Figure 1.**
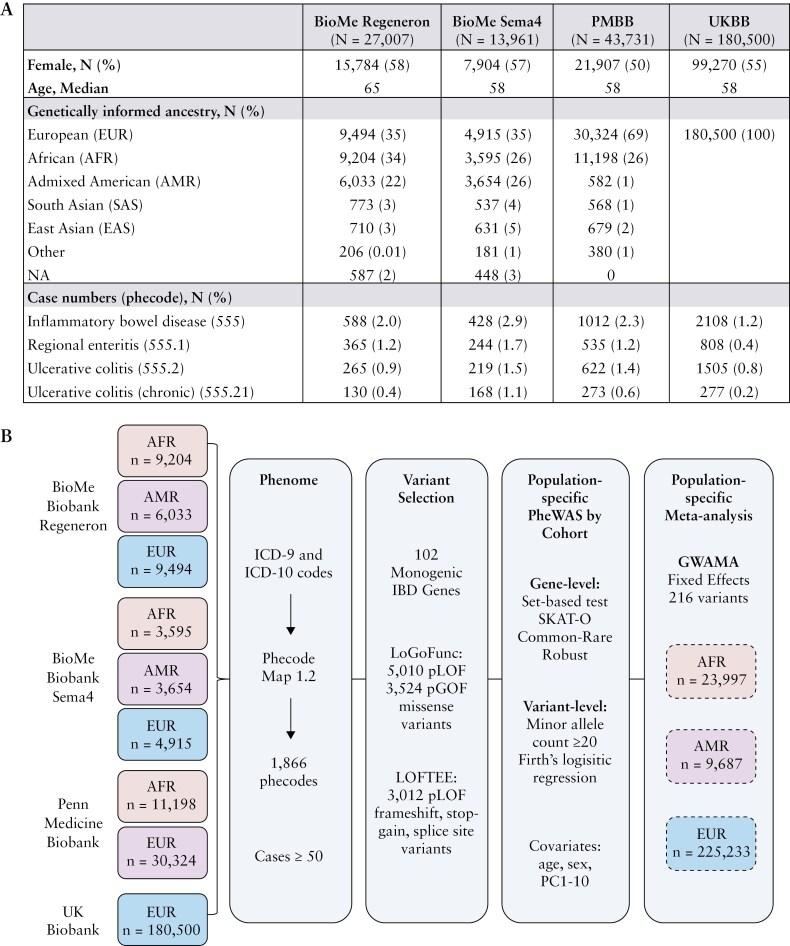
Overview of cohorts and PheWAS study workflow. (A) Demographic characteristics, including number of IBD cases, are summarized by cohort. (B) The study population consists of individuals genetically similar to African (AFR), Admixed American (AMR), and European (EUR) populations from 4 cohorts with whole exome sequencing and phenotypic data. ICD-9 and ICD-10 codes were mapped to phecodes using Phecode Map 1.2. Predicted loss-of-function (pLOF) and gain-of-function (pGOF) variants were extracted from 102 monogenic IBD genes using LoGoFunc and LOFTEE methods. Population-specific phenome-wide association analyses (PheWAS) were conducted at the gene and variant level for each cohort and assessed for replication. Population-specific fixed-effect meta-analysis of PheWAS results was performed for variants and phenotypes that passed quality control criteria in at least 2 cohorts.

BioMe BioBank (Regeneron and Sema4 datasets) and PMBB are prospective hospital-based biobanks ascertained generally, which comprise electronic health records of participants linked to WES data (https://icahn.mssm.edu/research/ipm/programs/biome-biobank, https://pmbb.med.upenn.edu) (Figure 1A). Patient diagnoses are documented using ICD-9 and ICD-10 codes, and these codes are subsequently translated into clinically meaningful phenotype codes (phecodes) using Phecode Map v1.2. Informed consent was obtained from all biobank participants. BioMe and PMBB participants display high levels of population diversity and were assigned to population groups by genetic similarity as detailed (Supplementary Methods).

The UKBB is a prospective study involving around 500 000 participants, including a diverse range of phenotypic outcomes (Figure 1A). In this study, we utilized the 200k exome release (Fields 23155 and 23156 in the UKBB Showcase). ICD-9 and ICD-10 codes were extracted and subsequently mapped to phecodes using Phecode Map v1.2. Informed consent of all participants was obtained by UKBB. WES processing and quality control assessment were performed as detailed (Supplementary Methods).

### 2.2. Gene selection, variant annotation, and identification of predicted LOF and GOF variants

We analyzed 102 genes previously established by Bolton et al. in a taxonomy of monogenic IBD. These genes were identified through an extensive literature review of reported monogenic disorders associated with IBD and supported by functional evidence.^12^ The reported cases primarily involved individuals with very early onset IBD and included patients of EUR and non-EUR ancestry. From the BioMe and UKBB cohorts, we extracted variants predicted to have a functional impact on proteins, including high-impact variants (stop gain, stop loss, frameshift, and essential splice site) and missense mutations, as annotated by Variant Effect Predictor (VEP). To predict the directionality of the impact of missense variants, we employed LoGoFunc, a machine-learning classifier we recently developed that reliably distinguishes pathogenic predicted LOF (pLOF) and predicted GOF (pGOF) variation.^13^ We also used LOFTEE, a VEP plugin tool, to identify pLOF variation from high-impact variants.^14^ Variant minor allele frequencies were retrieved from gnomAD exomes v4.1.0 using the gnomADe VEP plugin.

### 2.3. Gene- and variant-level PheWAS

We performed gene-level PheWAS by collapsing all identified pLOF and pGOF variants in monogenic IBD genes in EUR, AFR, and AMR population groups in each cohort when present (Figure 1B).

For the gene-level PheWAS, we employed the SKAT-O Binary robust method, maintaining a case-control ratio of 1:99.^15^ Phecodes with a minimum of 50 cases were included in the analysis. Age, sex, and principal components (PCs) 1-10 were included as covariates in the analysis.

We selected pLOF and pGOF variants with a minor allele count (MAC) > 20 in each population group and conducted variant-level PheWAS using Firth’s logistic regression method, implemented in Plink v2.^16^ Similar to the gene-level analysis, phecodes with a minimum of 50 cases and the same covariates (age, sex, and PCs1-10) were included in the analysis. Associations with at least 2 carrier cases were retained to enhance result robustness, especially addressing issues originating from low allele frequencies and skewed case-control ratios.

Study-wide significance thresholds were determined by adjusting for multiple tests using the Benjamini-Hochberg False Discovery Rate (FDR) method. Gene- and variant-level associations were considered study-wide significant at a threshold of FDR *P* < 0.1, as has been used in prior PheWAS studies.^17^ Given the rarity of variants analyzed, we also reviewed all associations that reached nominal significance (*P* < .05). An association was considered replicated if it reached at least nominal significance (*P* < .05) for the same phecode or its corresponding parent phenotype (integer level of the phecode) in an independent cohort of the same population group. For example, “Regional enteritis” (555.1) and “Ulcerative colitis” (555.2) are both nested under the parent phecode “Inflammatory bowel disease” (555). Further, for variant-level PheWAS, associations were only considered replicated if odds ratios had the same direction of effect across cohorts.

### 2.4. Variant-level population-specific PheWAS meta-analysis

GWAMA was employed to conduct a population-specific fixed-effects meta-analysis in AFR (BioMe and PMBB), AMR (BioMe), and EUR (BioMe, PMBB, and UKBB) groups (Figure 1B). Variants and phenotypes that passed QC in at least 2 cohorts were included in meta-analyses. *P* values were adjusted according to genomic control values for each phenotype.

### 2.5. Single-cell RNA sequencing

Samples for single-cell RNA sequencing from Martin et al. and Levantovsky et al. were integrated using Harmony v0.1.0.^18,19^ Briefly, samples from Martin et al. included inflamed and non-inflamed mucosal biopsies and peripheral blood mononuclear cells (PBMCs) from patients with CD undergoing ileal resection and were processed using the 10× Chromium Single Cell 3’ Library & Gel Beat Kit v2.^18^ Libraries were sequenced using Illumina NextSeq500. Samples from Levantovsky et al were obtained from inflamed distal rectum and perianal fistula tracts from patients with CD undergoing completion proctectomy and processed using the 10x Chromium Next GEM Single Cell 3’ Kit v3.1.^19^ Libraries were sequenced on Illumina NovaSeq 6000. Clustering was performed using Seurat v4.2.0.

## 3. Results

### 3.1. Variant overview

We extracted 11 546 pLOF and pGOF variants in 102 monogenic IBD genes. Over two-thirds of the variants (*n* = 8022) were predicted to be LOF by LOFTEE and LoGoFunc (Figure S1C). Of the variants reported in gnomAD exomes v4.1.0 (*n* = 6868), most were rare with a minor allele frequency (MAF) ≤ 0.1% (*n* = 6815) with 6382 (93%) being ultra-rare with MAF ≤ 0.01% (Table S1 and Figure S1A). A total of 1172 variants were reported to exhibit at least 10-fold enrichment in individuals of AFR ancestry compared to Non-Finnish EUR (NFE) ancestry in gnomAD v.4.1.0, with higher numbers of AFR-predominant variants found in BioMe and PMBB (Figure S1B).

### 3.2. Gene-level analyses replicate IBD associations in genes within previously reported GWAS loci and include shared and unique genetic factors in IBD across populations

**Figure 2.**
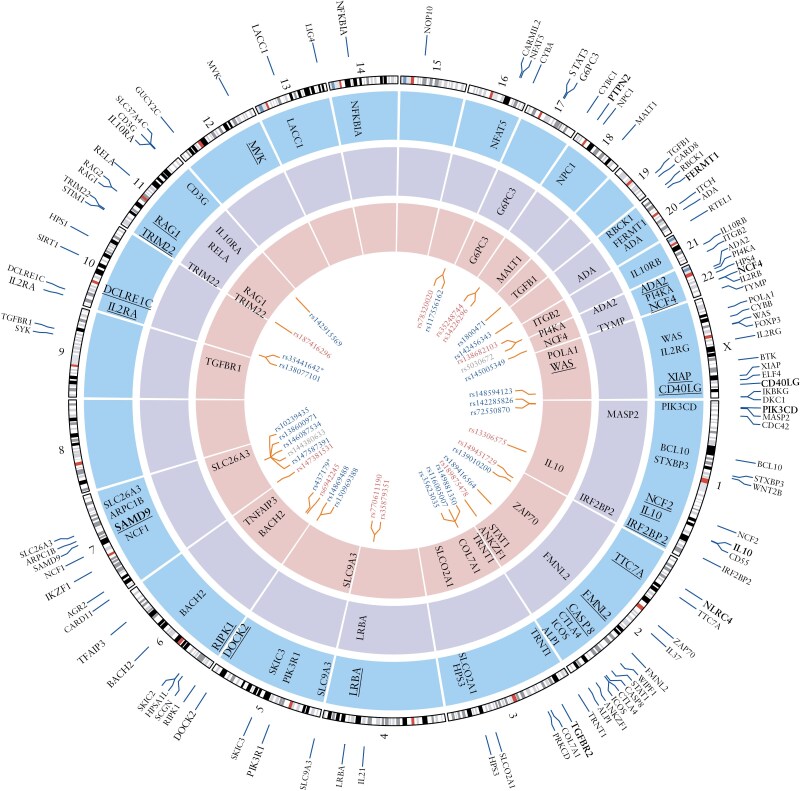
Gene- and variant-level analyses identifies shared and population-specific IBD genetic factors in monogenic IBD genes. The outermost track of the circos plot displays 102 genes reported to cause monogenic IBD (genes in bold overlap with known IBD GWAS loci). Inner tracks show gene-level associations with IBD phenotypes (phecodes 555, 555.1, 555.2, and 555.21) in 3 populations: European (blue layer), Admixed American (purple layer), and African (red layer). Genes shown in bold and underlined represent associations with IBD phenotypes that occurred in at least 2 independent cohorts within the same population group (*P* < .05). The innermost track highlights variants associated with IBD phenotypes in population-specific PheWAS meta-analysis (*P* < .05). Text color denotes population-specific associations: red (African), blue (European), and gray (both populations). All associations indicate increased risk for IBD except variants marked with an asterisk (*).

Of the 102 monogenic genes, 20 overlap with polygenic IBD risk loci implicated in prior GWAS or reported in large-scale sequencing studies^1,2,20–24^ (Figure 2, bolded in the outermost ring of gene names). We considered IBD phenotypes as any under the parent phecode 555 (Inflammatory bowel disease), which includes phecodes 555.1 (Regional enteritis), 555.2 (Ulcerative colitis), and 555.21 (Ulcerative colitis—chronic). We detected nominal IBD associations (*P* < .05) in 15 of the GWAS overlapping genes, with 5 genes, including *IL10*, *DOCK2*, *IL2RA*, *NCF4*, and *CD40LG* showing evidence of replication in independent EUR cohorts (Figure 2, bolded blue ring and Table S2). Beyond these 5 genes, EUR-replicated rare-variant, gene-level associations not overlapping GWAS loci were observed in an additional 14 genes; these 14 genes include well-known pathways related to IBD,^12^ such as *XIAP* (downstream of *NOD2*). EUR cohorts revealed the highest number of genes with nominal IBD associations (47), followed by AFR (22) and then AMR cohorts (11), reflecting the relative power of the cohorts. Seventeen genes, including *ADA*, *LRBA*, and *TRIM22* showed associations across population-specific cohorts, indicating shared genetic architecture even for rare variant-driven gene associations (Figure 2 and Table S2). Notably, associations in genes, such as *ITGB2*, were only observed in AFR cohorts. *TRIM22* was linked to IBD phenotypes across all 3 population groups (Figure 2).

### 3.3. Variant-level analyses reveal population-specific allelic heterogeneity of IBD risk genes and AFR-predominant variants

We next performed variant-level PheWAS for variants surpassing a MAC threshold of 20 to investigate IBD associations of individual variants located in monogenic IBD genes and differentially assess the effects of pLOF and pGOF variants. The number of variants analyzed per group was as follows: 168 (Regeneron AFR), 140 (Regeneron EUR), 135 (Regeneron AMR), 111 (Sema4 AMR), 107 (Sema4 EUR), 121 (Sema4 AFR), 217 (PMBB EUR), 170 (PMBB AFR), and 647 (UKBB). We identified 27 variants across 20 monogenic genes with IBD-related associations that met the study-wide significance of FDR-adjusted *P* < .1 (Table S3), primarily in UKBB. Some of these FDR-significant associations exclusively observed in UKBB reflect extremely rare associations, such as pGOF (rs201778935) in *CTLA4*; given the absence of gene-based or GWAS associations (Figure 2), this gene cannot be considered as replicated in our present, biobank cohorts. Similar, single allele EUR-predominant associations drive the *HPS3* and *RAG2* associations, which therefore cannot be considered replicated. A replicated association was observed for a rare pLOF missense variant in *SKIC3* (rs146627706; MAF 0.04%), which increased the risk of IBD and UC at FDR adjusted *P* < .1 in both UKBB and the EUR group in PMBB (Table S3).

Rare pLOF variants in *TRIM22* (regulator of NOD2 signaling) have been implicated in severe forms of VEO-IBD, characterized by granulomatous colitis and perianal fistulizing disease.^25,26^ One of the reported missense variants, rs187416296,^25^ was nominally associated with increased risk of IBD across all population groups in the Regeneron cohort (Table 1). Additionally, previously unreported rare pLOF variants were associated with IBD in UKBB. Similarly, pLOF, distinct (likely EUR-dominant) alleles in *NPC1* were reported by the Uhlig lab as causing primarily granulomatous colitis.^27^ Pathogenic variants in *NPC1* cause Niemann-Pick type C (NPC) disease which is an autosomal recessive, progressive, and often fatal disease affecting nerve and muscle cells resulting from impaired intracellular cholesterol transport and lipid accumulation within cells. Chiorean et al. explored the 6 major population groups in gnomAD (*n* = 122 678) for the pLOF variants in *NPC1* and observed marked population differences in frequencies of pLOF variants in *NPC1*, with AFR-Americans having the highest carriage (3.26%).^28^ Consistently, we observed enrichment of AFR-predominant pLOF *NPC1* alleles in our cohorts in association with IBD at the variant level and AFR-specific meta-analysis (Table 1).

**Table 1.** Inflammatory bowel disease-associated risk variants predominant in African populations.

Gene	Variant	MAF AFR	MAF NFE	Direction	Phenotype	Variant-level PheWAS			Meta-analysis		
						FDR adj *P* < .1	*P* < .01	*P* < .05	OR	*P*	Ancestry
ADA	rs142456343	0.00000	0.00003	pLOF	Regional enteritis		Reg EUR		**3.424**	**.010**	EUR
					Inflammatory bowel disease				**2.166**	**.032**	EUR
	rs61732239	0.01324	0.00008	pLOF	Inflammatory bowel disease		Reg AMR	Reg AFR	1.595	.197	AFR
					Ulcerative colitis		Reg AFR		2.639	.079	AFR
	rs121908736	0.00329	0.00002	pLOF	Ulcerative colitis			PMBB AFR	3.948	.117	AFR
ADA2	rs77563738	0.00000	0.00057	pLOF	Ulcerative colitis		UKBB			nt	
	rs115986203	0.01568	0.00002	pLOF	Inflammatory bowel disease		Reg AMR	Sema4 AFR	1.727	.204	AFR
CARD11	rs201847585	0.00000	0.00020	pGOF	Ulcerative colitis	UKBB				nt	
					Inflammatory bowel disease		UKBB			nt	
	rs147381531	0.00323	0.00030	pGOF	Regional enteritis		PMBB AFR		**3.908**	**.045**	AFR
FMNL2	rs189416564	0.00021	0.00180	pLOF	Regional enteritis	UKBB			**2.575**	**.014**	EUR
					Inflammatory bowel disease		Reg AMR	UKBB	**1.705**	**.046**	EUR
	rs142575649	0.00772	0.00000	pLOF	Ulcerative colitis			Reg AFR	3.025	.145	AFR
	rs34119671	0.02959	0.00002	pLOF	Regional enteritis			PMBB AFR	1.871	.051	AFR
ITGB2	rs778653538	0.00000	0.00014	pGOF	Inflammatory bowel disease	UKBB				nt	
					Ulcerative colitis			UKBB		nt	
	rs141799330	0.01171	0.00006	pLOF	Inflammatory bowel disease			Sema4 AFR	1.945	.251	AFR
	rs138682103	0.00356	0.00000	pLOF	Inflammatory bowel disease			Sema4 AFR	2.254	.148	AFR
					Regional enteritis			PMBB AFR	**3.804**	**.027**	AFR
LRBA	rs200802435	0.00003	0.00016	pLOF	Inflammatory bowel disease	UKBB				nt	
					Regional enteritis	UKBB				nt	
					Ulcerative colitis		UKBB			nt	
	rs35250375	0.01102	0.00002	pLOF	Regional enteritis		Reg AFR		2.377	.068	AFR
	rs148385798	0.00633	0.00000	pLOF	Regional enteritis			PMBB AFR	2.864	.071	AFR
NCF2	rs147415774	0.00027	0.00163	pLOF	Inflammatory bowel disease	UKBB				nt	
					Ulcerative colitis	UKBB				nt	
					Ulcerative colitis (chronic)	UKBB				nt	
					Regional enteritis	UKBB				nt	
	rs13306575	0.00117	0.00004	pLOF	Ulcerative colitis		PMBB AFR		**5.499**	**.024**	AFR
NPC1	rs34302553	0.01027	0.00033	pLOF	Inflammatory bowel disease	UKBB			1.793	.104	AFR
					Ulcerative colitis	UKBB			1.712	.339	AFR
					Ulcerative colitis (chronic)	UKBB				nt	
	rs34226296	0.00956	0.00000	pLOF	Inflammatory bowel disease		Sema4 AFR		**2.403**	**.025**	AFR
					Regional enteritis				**3.529**	**.011**	AFR
	rs35248744	0.01293	0.00000	pLOF	Regional enteritis		PMBB AFR		**2.547**	**.049**	AFR
	rs13381670	0.00971	0.00001	pLOF	Inflammatory bowel disease			PMBB AFR	2.004	.099	AFR
					Ulcerative colitis			PMBB AFR	2.630	.079	AFR
SAMD9	rs144380633	0.00314	0.01027	pGOF	Regional enteritis	UKBB		PMBB AFR	**1.709**	**.005**	EUR
					Inflammatory bowel disease		PMBB AFR	Reg EUR	**3.164**	**.041**	AFR
					Ulcerative colitis			PMBB AFR	1.689	.130	AFR
					Ulcerative colitis (chronic)			Reg EUR	1.162	.725	EUR
	rs748338739	0.00000	0.00006	pGOF	Inflammatory bowel disease	UKBB				nt	
					Ulcerative colitis	UKBB				nt	
	rs138478808	0.00048	0.00026	pGOF	Inflammatory bowel disease	UKBB				nt	
					Regional enteritis		UKBB			nt	
					Ulcerative colitis		UKBB			nt	
	rs115350620	0.01219	0.00000	pGOF	Inflammatory bowel disease			Reg AMR	1.628	.240	AFR
					Regional enteritis			PMBB AFR	2.470	.071	AFR
	rs76377166	0.04505	0.00003	pGOF	Inflammatory bowel disease			Sema4 AFR	1.689	.130	AFR
TRIM22	rs199731307	0.00003	0.00013	pLOF	Regional enteritis	UKBB				nt	
					Inflammatory bowel disease		UKBB			nt	
	rs371728648	0.00000	0.00009	pLOF	Inflammatory bowel disease	UKBB				nt	
					Ulcerative colitis	UKBB				nt	
	rs187416296	0.00093	0.00803	pLOF	Inflammatory bowel disease		Reg AFR, Reg AMR	Reg EUR	**6.074**	**.003**	AFR
					Ulcerative colitis		Reg AFR		**7.660**	**.022**	AFR
					Regional enteritis		Reg EUR		**6.052**	**.029**	AFR

Inflammatory bowel disease phenotypes were defined using phecodes 555 (Inflammatory bowel disease), 555.1 (Regional enteritis), 555.2 (Ulcerative colitis), and 555.21 (Ulcerative colitis [chronic]). Allele frequencies of variants enriched at least 10-fold in African populations are highlighted in gray. Allele frequencies were obtained from gnomAD v4.1.0. Nominally significant results (P < 0.05) in Meta-analysis are highlighted in bold.

MAF: minor allele frequency, AFR: African, NFE: Non-Finnish European, EUR: European, AMR: Admixed American, pLOF: loss-of-function, pGOF: gain-of-function, OR: odds ratio, FDR: false discovery rate, adj: adjusted, PheWAS: phenome-wide association study, nt: not tested.

We further sought to examine variants enriched at least 10-fold in AFR ancestry individuals compared to NFE ancestry individuals in non-UKBB cohorts. We focused on genes that also harbored variants with study-wide significant associations in EUR variant-level analyses. For instance, a pGOF variant in *CARD11* (rs147381531) with 0.3% MAF in AFR ancestry and 0.03% MAF in NFE ancestry was associated with an increased risk of regional enteritis in AFR group analyses in PMBB (Table 1). Further, heterozygous carriage of Goldilocks AFR-predominant pLOF variants in *ADA* and *ADA2* were associated with increased risk of IBD and UC in BioMe and PMBB (Table 1). *ADA* and *ADA2* both encode adenosine deaminase enzymes involved with purine metabolism, albeit with differences in localization and function. *ADA* is ubiquitously expressed and functions intracellularly to deaminate adenosine, while *ADA2* is primarily secreted by myeloid cells and plays signaling roles that are not yet fully understood.^29,30^ Phenotypically, rare pLOF *ADA* variants have been implicated in immunodeficiencies (including severe combined immunodeficiency), while *ADA2* deficiency is associated with systemic autoinflammation with vasculitis and mild immunodeficiency.^31,32^

### 3.4. Gene- and variant-level PheWAS and meta-analysis reveal associations with other, non-IBD phenotypes

Genetic defects implicated in monogenic IBD often lead to extraintestinal manifestations, including recurrent infections, dermatologic abnormalities, autoimmunity, hematologic aberrations, malignancy, and hepatic abnormalities.^9^ Therefore, we explored phenotypic associations other than IBD in the PheWAS results, revealing a total of 586 variant-level associations (544, EUR, 40 AFR, and 2 AMR) meeting study-wide significance (FDR adjusted *P* < .1) and replication (Table S5). As a validation example, we examined the well-established association between the pGOF *JAK2* rs77375493 (V617F) allele and the increased risk of myeloproliferative disorders, confirming this link across cohorts (Figure S2). Among the monogenic IBD genes, the strongest replicated association observed at the variant level was the previously reported link between the pGOF *CTLA4* variant (rs231775) and increased risk of autoimmune thyroid disorders across all cohorts (Tables S5). On meta-analysis, this variant was associated with increased risk of hypothyroidism at study-wide significance in both EUR and AMR groups, while nominally associated in the AFR group at *P* < .05 (Tables S6–S8). Gene- and variant-level associations that replicated across populations included phenotypes more commonly reported in monogenic IBD, including infection (viral and bacterial), autoimmunity (hypothyroidism, psoriasis, inflammatory spondyloarthropathies), and hepatobiliary phenotypes (hepatomegaly, cholelithiasis) (Tables S4 and S5). Notably, cardiac dysrhythmias and conduction disorders were the most frequently replicated associations, suggesting possible pleiotropy between monogenic IBD genes and cardiac rhythm disorders.

Population-specific analyses revealed multiple gene- and variant-trait associations specific to AFR groups in BioMe and PMBB cohorts. These effects were primarily observed in variants enriched in AFR populations and ultra-rare in EUR groups. The strongest replicated gene- and- variant-level association was observed with *TRIM22* and increased risk of “Hereditary hemolytic anemias” and “Sickle cell anemia” in Regeneron and PMBB (Tables S4 and S5). A meta-analysis confirmed associations between these phenotypes and 2 pLOF variants in *TRIM22* (rs61735328 and rs114191522) in the AFR group (Table S6). Because *TRIM22* is in the vicinity of *HBB on* chromosome 11, we investigated whether these associations were conditionally dependent on pathogenic variants in *HBB.* We conducted a conditional analysis in the Regeneron AFR cohort using pathogenic and likely pathogenic *HBB* variants reported in gnomAD based on ClinVar annotations (Table S9), which abolished the significant association of the *TRIM22* variants with “Hereditary hemolytic anemias” and “Sickle cell anemia” (Table S10). These results suggest that the observed associations of these pLOF variants in *TRIM22* with hemolytic anemia and sickle cell anemia are driven by linkage disequilibrium with pathogenic *HBB* variants. Notably, these variants were distinct from other *TRIM22* pLOF variants associated with IBD (Table S3).

**Figure 3.**
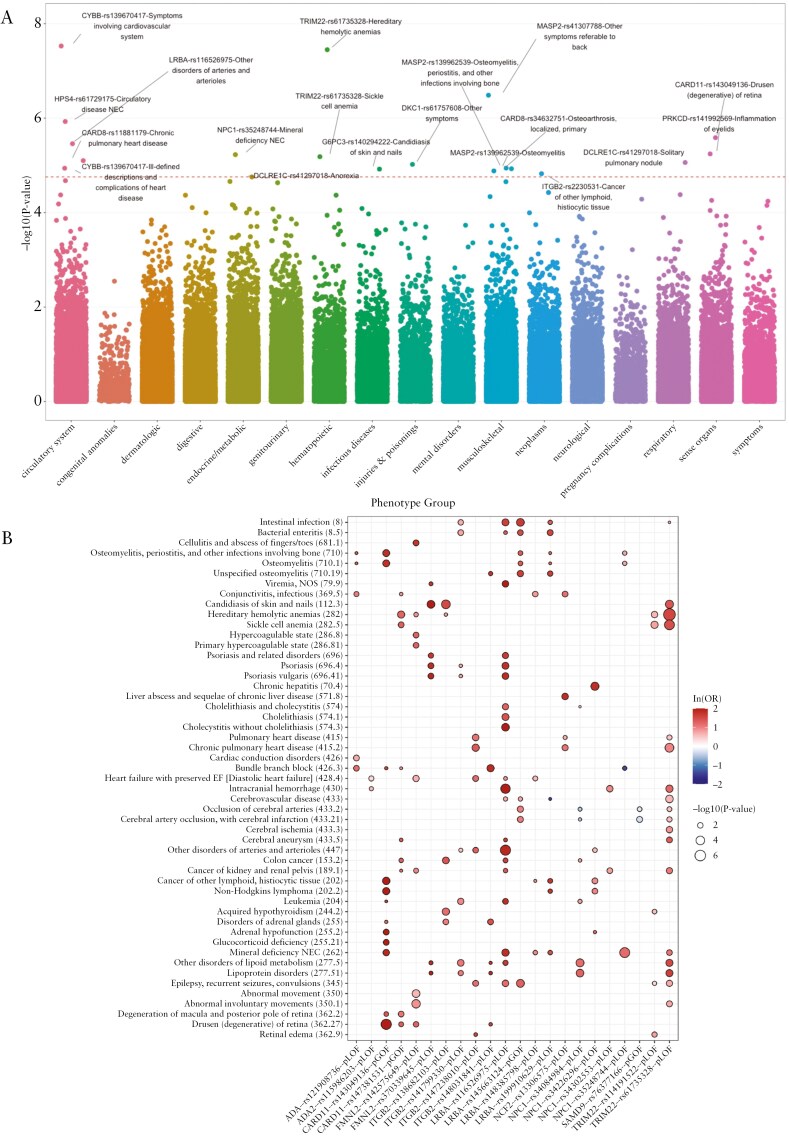
Meta-analysis of variant-level PheWAS in African populations in BioMe and Penn Med Biobank. (A) Manhattan plot of variant-phenotype associations. The red dashed line represents a study-wide significance level of false discovery rate adjusted *P* < .1. (B) Select phenotype associations reaching at least nominal significance of *P* < .05 in genes with AFR-predominant variant level IBD associations (see Table 1).

A pGOF *CARD11* variant (rs147381531), which was associated with IBD, was also linked to an increased risk of “Hereditary hemolytic anemias” in AFR groups in Regeneron and PMBB, with AFR-specific meta-analysis showing significance at *P* < .01 (Figure 3B and Table S6). pGOF variants in *CARD11* are known to cause immunodeficiency characterized by B-cell expansion and constitutive NF-κB activation.^33,34^ While hemolytic anemia has not been reported in this syndrome, somatic GOF mutations in *CARD11* have been reported in patients with cold agglutinin disease, resulting in autoantibody-mediated hemolytic anemia.^35^  *CARD11* pGOFs (rs143049136, rs147381531) also showed replication of macular degeneration at the variant level and confirmed on meta-analysis in the AFR group (Figure 3A and B and Table S6).

Several monogenic IBD genes extend their function beyond cytokine signaling, impacting processes such as immunometabolism and vesicle trafficking. *ADA* (adenosine deaminase) was linked to “Cardiac conduction disorders” at the gene level and variant level in a pLOF AFR-enriched variant rs121908736 in AFR groups in Regeneron and PMBB. This association was observed specifically in the AFR group meta-analysis (Figure 3 and Table S6). Adenosine, a key regulator of cardiovascular function, exerts its effects through mechanisms, including atrioventricular nodal blockade, and is used as a first-line treatment for supraventricular tachycardia.^36^ Impaired *ADA* function, leading to elevated adenosine levels, may amplify these receptor-mediated effects, contributing to the observed cardiac conduction phenotypes in individuals carrying this variant.

Further, pLOF variants in *NPC1* were associated with an increased risk of “Lipoprotein disorders” (rs34084984) and “Mineral deficiency” (rs35248744) in meta-analysis in the AFR group (Figure 3A and B and Table S6). This is, perhaps, not surprising given *NPC1*’s role in cholesterol and long-chain fatty acid metabolism and kinetics. Heterozygous carriage of pLOF *NPC1* alleles, which is highest in African individuals, are at increased risk for obesity, especially when combined with a high-fat diet.^28,37^ Chiorean et al showed no evidence for negative selection; rather, the authors speculated that balancing selection (ie, heterozygote advantage) of these AFR-predominant, pLOF variants in *NPC1* may have occurred, possibly reflecting evolutionary metabolic needs.

### 3.5. Expression of monogenic IBD genes in single-cell data

**Figure 4.**
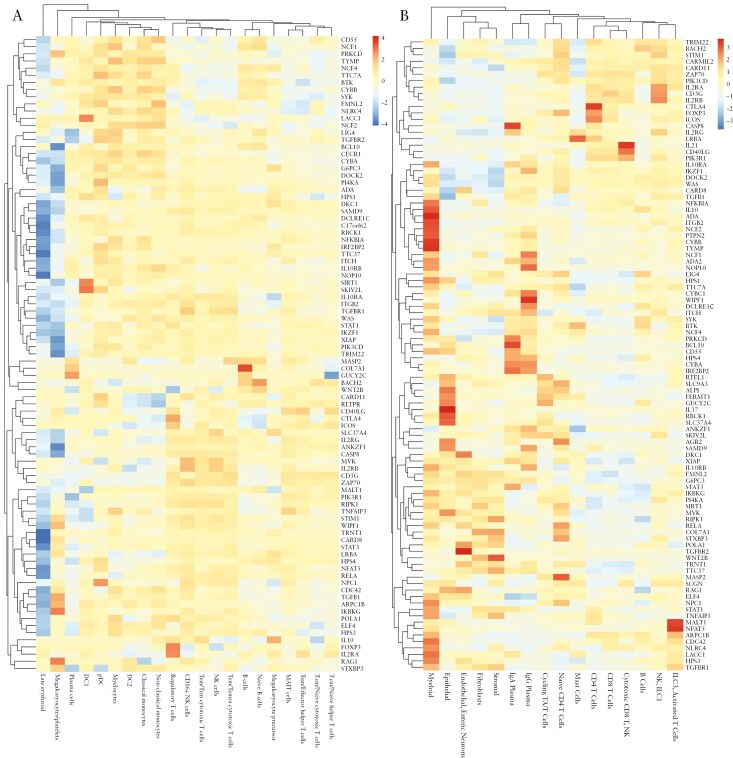
Monogenic IBD gene expression in peripheral blood and ileal and colonic tissue. Heatmap of mean expression by cell type cluster as sorted by Seurat v4.2.0 in (A) peripheral blood mononuclear cells and (B) ileal and colonic tissue from patients with Crohn’s disease. Genes and cell type clusters were grouped by gene expression similarity. *ADA2* is annotated as *CECR1* in peripheral blood.

Single-cell sequencing from PBMCs and ileal and colonic tissue from CD patients revealed distinct expression patterns of monogenic IBD genes across cellular compartments (Figure 4). These patterns were consistent across major immune subsets (myeloid and T cell, B cell) in blood and tissue. For example, genes, such as *CTLA4* and *FOXP3*, showed high expression in the regulatory T cell cluster in PBMCs and in CD4 T cell subsets in tissue. Epithelial-specific genes, including *AGR2, ALPI, FERMT1, IL37*, and *RTEL1*, were exclusively detected in tissue. Approximately two-thirds of monogenic IBD genes show strong expression in myeloid subsets. Genes with the highest expression in myeloid subsets in tissue, such as *NCF1, NCF2*, and *ITGB2,* showed variant-level associations with IBD. Other genes with IBD-associated variants, including *NPC1*, *SCGN*, and *TRIM22,* displayed broader expression patterns across myeloid, stromal, and lymphocyte populations. *ADA* was uniformly expressed in myeloid, T cell, and plasma cell clusters in PBMCs but showed stronger expression in myeloid cells in tissue. Meanwhile, *ADA2* (annotated as *CECR1* in PBMCs), previously reported to be induced during monocyte-to-macrophage differentiation,^38^ was expressed in myeloid and plasma cells in both blood and tissue. These distinct expression profiles suggest that adenosine deaminases may have context-dependent effects on CD pathogenesis.

LOF variants in components of the nicotinamide adenine dinucleotide phosphate (NADPH) oxidase complex, including epithelial *NOX1*, impair reactive oxygen species generation, leading to dysregulated immune response and compromised gut mucosal barrier integrity.^39,40^ Heterozygous variants in phagocytic NADPH oxidase components have also been implicated in very early onset IBD without apparent immunodeficiency.^39^ These findings suggest variants in monogenic IBD genes may influence cell-specific inflammatory processes driving CD pathogenesis, with enriched expression in myeloid cells highlighting the critical role of innate immunity in intestinal homeostasis. Interestingly, multiple monogenic IBD genes are expressed in platelets in the PBMC data. Platelets are not well represented in tissue-based data, which was generated after cell dissociation, but they may play important roles upon activation, contributing to proinflammatory cytokine release and endothelial dysfunction.

## 4. Discussion

In this study, we leverage diverse cohorts with WES data to investigate the role of monogenic IBD genes and their variants in IBD susceptibility and a broad spectrum of phenotypes. Monogenic forms of IBD are primarily driven by rare, highly penetrant mutations in genes affecting cellular compartments throughout the gut mucosal layers, maintaining intestinal homeostasis.^12^ We systematically defined the overlap between monogenic IBD genes and GWAS loci (common variants of often lower effect), demonstrating that common and rare variations converging on shared biological pathways contribute to shared phenotypes. Importantly, we define population-specific effects, where associations found with AFR-enriched variants would not have been discovered in EUR-focused cohorts alone, highlighting the importance of the inclusion of diverse populations in genomic studies.

We observed overlap in multiple genes with variant-level IBD associations in EUR and AFR cohorts, suggesting shared genetic architecture but with population-specific allele effects driven by variation in allele frequency. We identified Goldilocks alleles enriched in AFR populations that may contribute to IBD risk that are ultra-rare or absent in EUR populations. Notably, 2 pLOF variants in *LRBA* (rs35250375, rs148385798), observed more frequently in individuals of AFR ancestry, showed nominal associations with IBD phenotypes in Regeneron and PMBB. Mutations in *LRBA* are known to cause primary immunodeficiency and autoimmunity in an autosomal recessive manner.^41^ Gettler et al. using BioMe, showed that carriers of the pLOF variant (rs151213445) exhibited significantly reduced expression of *CTLA4* and *LRBA* upon T cell activation, indicating that these variants may increase disease risk even with only heterozygous carriage.^11^  *LRBA* assists with the recycling of *CTLA4* to the cell surface, so pLOF variants would be predicted to alter regulatory to effector lymphoid cell ratios; in gut tissues, LRBA is expressed in numerous T cell subsets plus mast cells (Figure 4B). Psoriasis shares many GWAS loci with IBD, notably along the IL23 pathway^42^ affecting lymphoid cells; given this, we note that pLOF *LRBA* alleles also confer the risk for psoriasis (Figure 4B). Multiple case reports have implicated rare *LRBA* variants being associated with the treatment of refractory psoriasis.^43^

Monogenic IBD genes are expressed in various cellular subtypes on single-cell RNA sequencing data from CD patients with roughly two-thirds highly expressed in myeloid cells (Figure 4). We suggest key pathways in myeloid cells contributing to IBD pathogenesis linking inflammation and cellular metabolism processes. Specifically, pLOF of *NPC1*, a well-studied intracellular cholesterol transporter, has been shown to impair autophagy, leading to granulomatous intestinal inflammation and perianal disease reminiscent of CD in autosomal recessive form.^27^ Heterozygous carriage of these variants has also been associated with an increased risk of obesity, with the highest allele frequencies observed in African populations.^28,37^ We identified AFR-predominant pLOF alleles in *NPC1*, which increased the risk of IBD and lipoprotein disorders. Emerging evidence suggests a role for dietary fatty acids, particularly long-chain, polyunsaturated fatty acids, in modulating intestinal inflammation. This highlights a potential avenue for dietary interventions that could influence the progression of IBD in the future. Adenosine deaminases (*ADA* and *ADA2*), which catabolize the purine nucleotide/-side (deoxy)-adenosine have been shown to correlate with disease activity in CD and UC.^44–46^ pLOF variants of *ADA* are known to cause severe combined immunodeficiency while *ADA2* is linked with a systemic autoinflammation disorder, suggesting important roles of adenosine signaling in modulating immune response and inflammation. The distinct single-cell expression patterns between *ADA* and *ADA2* and the markedly distinct rare pLOF variants between EUR ancestry and AFR populations may contribute to population differences in pathomechanisms of CD.

Our study is not without limitations. The smaller sample sizes in AFR and especially AMR cohorts reduce the statistical power to detect and replicate associations, particularly given the predominance of ultra-rare variants with pLOF and pGOF effects. Further, while our study identified novel IBD associations of pLOF and pGOF variants, functional validation is required to elucidate their precise roles in IBD pathogenesis. Our computational predictions for the directionality of impact ultimately require experimental corroboration to confirm mechanism and pathogenicity. Additionally, all the biobanks included in this study primarily consist of adult patients, while a significant proportion of IBD cases manifest during childhood. Including pediatric patients in future large-scale biobank efforts are essential, as children may present distinct disease mechanisms not captured in adult cohorts. Expanding large-scale studies to include younger populations could provide additional insights into the early onset of IBD and its genetic drivers.

Importantly, in this study, we link monogenic IBD genes with primarily adult-onset AFR-predominant Goldilocks alleles of epidemiologic (ie, not ultra-rare) and statistical significance even with the heterozygous carriage. If estimated effect sizes (Table 1) are generally replicated, these associations would be of frequent clinical significance. The incidence of IBD is rising in non-EUR populations, and these groups have been historically underrepresented in IBD GWAS; this results in gaps in our understanding of population-specific genetic contributions to IBD. We urge equitable representation in genomics research, which is critical to advancing risk prediction and improving outcomes for all IBD patients.

## Supplementary Material

jjaf098_suppl_Supplementary_Figures_1-2_Tables_1-10

## Data Availability

Functional predictions for all missense variants generated with LoGoFunc are available at https://itanlab.shinyapps.io/goflof/. Single-cell RNA sequencing data generated by Martin et al. and Levantovsky et al. are publicly available in GEO with the accession numbers GSE134809 and GSE260842 respectively.
