# Supplementary material for: Phenome-wide association study of monogenic inflammatory bowel disease genes in diverse biobanks identifies population-specific and shared Goldilocks alleles: implications for Precision Medicine
Source: J Crohns Colitis. 2025 Aug 5;19(7):jjaf098. doi: 10.1093/ecco-jcc/jjaf098 (PMC13223577; doi:10.1093/ecco-jcc/jjaf098)
Supplement: jjaf098_suppl_Supplementary_Figures_1-2_Tables_1-10 [file jjaf098_suppl_supplementary_figures_1-2_tables_1-10.zip › jjaf098_Suppl_Methods_Tables 1-10_Figures 1-2/Suppfigure1.pdf]

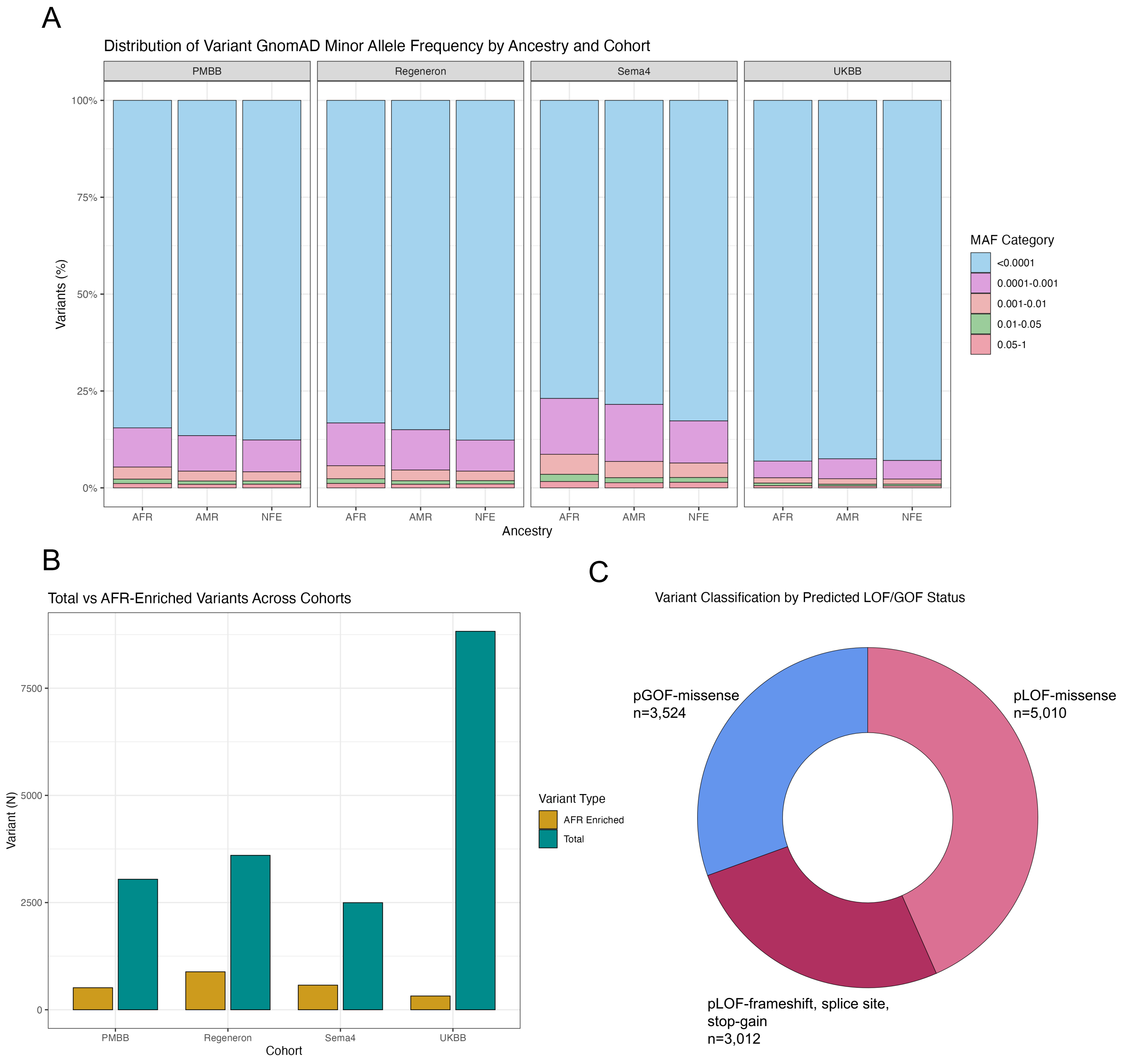
A
Distribution of Variant GnomAD Minor Allele Frequency by Ancestry and Cohort
PMBB
Regeneron
Sema4
UKBB
100%
75%
50%
25%
0%
Variants (%)
AFR
AMR
NFE
Ancestry
MAF Category
<0.0001
0.0001-0.001
0.001-0.01
0.01-0.05
0.05-1
B
Total vs AFR-Enriched Variants Across Cohorts
7500
5000
2500
0
Variant (N)
PMBB
Regeneron
Sema4
UKBB
Cohort
Variant Type
AFR Enriched
Total
C
Variant Classification by Predicted LOF/GOF Status
pGOF-missense
n=3,524
pLOF-missense
n=5,010
pLOF-frameshift, splice site,
stop-gain
n=3,012
