# Supplementary material for: Phenome-wide association study of monogenic inflammatory bowel disease genes in diverse biobanks identifies population-specific and shared Goldilocks alleles: implications for Precision Medicine
Source: J Crohns Colitis. 2025 Aug 5;19(7):jjaf098. doi: 10.1093/ecco-jcc/jjaf098 (PMC13223577; doi:10.1093/ecco-jcc/jjaf098)
Supplement: jjaf098_suppl_Supplementary_Figures_1-2_Tables_1-10 [file jjaf098_suppl_supplementary_figures_1-2_tables_1-10.zip › jjaf098_Suppl_Methods_Tables 1-10_Figures 1-2/Supplementary figure legends.docx]

**Supplementary Figure Legends**

**Supplementary Figure 1. Distribution of variant minor allele frequency and functional directionality.**

(A) and (B) depict the enrichment of African-predominant Goldilocks alleles in BioMe and Penn Med BioBank. (B) Variants are classified as African-enriched if their minor allele frequency is at least 10-fold higher in African populations compared to Non-Finnish Europeans, as reported in gnomAD v4.1.0. (C) High confidence loss-of-function variants were identified using LOFTEE, while missense variants were classified as loss-of-function and gain-of-function by LoGoFunc.

AFR: African, AMR: Admixed American, NFE: Non-Finnish European, MAF: minor allele frequency, LOF: loss-of-function, GOF: gain-of-function

**Supplementary figure 2. Variant-level PheWAS of JAK2 pGOF variant rs77375493 (V617F).**

Manhattan plots of associations in (A) UK Biobank and (B) BioMe Biobank Regeneron. All labeled associations indicate increased risk. Associations with myeloproliferative disease and polycythemia were replicated in the Penn Medicine Biobank. The variant did not meet the minor allele count threshold for testing in the BioMe Biobank Sema4 cohort.
