# Supplementary material for: Phenome-wide association study of monogenic inflammatory bowel disease genes in diverse biobanks identifies population-specific and shared Goldilocks alleles: implications for Precision Medicine
Source: J Crohns Colitis. 2025 Aug 5;19(7):jjaf098. doi: 10.1093/ecco-jcc/jjaf098 (PMC13223577; doi:10.1093/ecco-jcc/jjaf098)
Supplement: jjaf098_suppl_Supplementary_Figures_1-2_Tables_1-10 [file jjaf098_suppl_supplementary_figures_1-2_tables_1-10.zip › jjaf098_Suppl_Methods_Tables 1-10_Figures 1-2/Supplementary Methods.docx]

**Supplementary Methods**

**Sequencing and quality control**

**BioMe Regeneron dataset**

Whole exome sequencing (WES) was performed by Regeneron (NY, USA) using the IDT xGen capture kit on an Illumina v4 HiSeq 2500 platform for a total of 31,250 samples. The raw reads were aligned to the GRCh38 reference genome using the Burrows-Wheeler Aligner (BWA)^1^. Variant calling, recalibration and genotyping were performed by following with best practices guidelines of GATK, using Picard tools (http://broadinstitute.github.io/picard/) and GATK v.3.5^2^. Over 99% of the samples exhibited coverage > 85% for the targeted bases at 20X or higher.

Normalization of insertion-deletion variants (indels) and the splitting of multi-allelic variants were conducted utilizing BCFtools^3^. A quality control (QC) assessment was carried out to eliminate contaminated, low-coverage, gender-discordant, genotype-exome discordant, and duplicate samples, resulting in the exclusion of 437 samples. Additionally, second degree or closer relatives detected by KING^4^ and samples without any phenotype data were removed. Genotypes with a genotype quality (GQ) < 20, depth (DP) < 8 or allelic balance (AB) < 0.30 or > 0.80 were set to missing. Variant sites exhibiting a missingness rate > 0.02 were excluded from the dataset. The final Regeneron dataset comprised 27,740 samples and 7,637,342 variants.

**BioMe Sema4 dataset**

WES was performed by the Sema4 laboratory, using the Illumina NovaSeq6000 sequencing system with 100 bp paired-end reads. The Agilent SureSelect QXT Human All Exon V7 targeting kit was utilized for 15,084 samples. The alignment, variant calling, and genotyping processes were performed as described above. ≥ 89.5% of the targeted bases were covered at 20x coverage or more.

Following a sample-level QC check, four samples were excluded due to genotype-exome discordance. Samples without any phenotype data and second degree or closer relatives were also removed. Genotypes with a GQ < 20, DP < 8 or AB < 0.30 or > 0.80 were set to missing. Variant sites exhibiting a missingness rate > 0.02 were excluded from the dataset. The final Sema4 dataset included 14,186 samples and 7,447,558 variants.

**PMBB dataset**

WES was conducted by Regeneron (NY, USA). Samples were processed using the custom IDT xGen v1 exome capture kit on the Illumina NovaSeq 6000 system. Read alignment, variant calling, and genotyping were performed using a WeCall variant caller. Sample and variant-level QC checks were performed as previously described^5^. Samples with low coverage, gender discordance, duplication, and high rates of heterozygosity were excluded. The final PMBB dataset included 43,731 samples.

**UKBB dataset**

WES data for 200,000 samples were obtained, resulting in a dataset comprising 200,643 samples and a total of 17,981,897 variants. Variant calling and genotyping steps have been previously described^6^. Both sample- and variant-level QC checks were performed to obtain a high-quality WES dataset.

Samples with a genotype missingness rate > 0.05, gender-discordant, and duplicate samples detected by KING were removed. Moreover, second-degree or closer relatives were excluded. Variants with a missingness rate > 0.2 and a deviation from Hardy-Weinberg equilibrium (HWE) at a significance level of < 1x10^-6^ were removed. The final UKBB dataset included 189,448 samples and 17,402,344 variants.

**Population group assignment**

We assigned participants to population groups by genetic similarity, utilizing TOPMed-imputed array genotyping data, specifically the Global Sequencing Array (GSA) and Global Diversity Array (GDA) for BioMe participants, and the GSA for PMBB participants^7^. The analysis incorporated the 1000 Genomes Project reference panel (primary release, build 38, 3202 samples) with superpopulation labels to facilitate genetic similarity assessments (<https://www.cog-genomics.org/plink/2.0/resources>)^8^.

To determine the optimal number of ancestral populations (K), cross-validation errors were calculated for K values ranging from 4 to 12 using ADMIXTURE^9^. Subsequently, GrafPOP was employed to assign samples to superpopulations^10^. The Frobenius distance was calculated, which served as a metric to compare each ADMIXTURE matrix (K) with the GrafPOP matrix. K=10 displayed the best fit based on identifying the smallest distance (the closest match between the two matrices). Genetically inferred classes were assigned to an ancestry group by identifying the 1000 Genomes Project reference samples that displayed the highest proportion in each class.  Identified population groups included African (AFR), Admixed American (AMR), East Asian (EAS), European (EUR), and South Asian (SAS). For downstream analyses, population groups with sufficient case numbers were included: EUR, AFR, and AMR in BioMe, and EUR and AFR in PMBB.

Given that the majority of the UKBB dataset includes participants genetically similar to EUR populations, we refined the dataset to specifically include these samples using ADMIXTURE. The ancestral contributions of participants were evaluated by comparing them to reference populations from the 1,000 Genomes Project database, namely Utah residents with northern and western European ancestry (CEU), East Asian (EAS), and Yoruba in Ibadan (YRI). Variants were pruned based on linkage disequilibrium (LD) using --indep-pairwise option of Plink v1.9 with a window size of 50, a step size of 5 and an r^2^ of 0.2^11^. Then, common variants were selected using a minor allele frequency (MAF) threshold of > 0.02. In the admixture analysis, the parameter K was set to 3, representing the expected number of major ancestral populations. 180,500 samples with a EUR fraction greater ≥ 0.8 were classified as genetically similar to EUR.

Population-specific analyses were conducted for individuals in the EUR group across all four cohorts, for individuals in the AFR group in three cohorts (Regeneron, Sema4, PMBB), and for individuals in the AMR group in two cohorts (Regeneron, Sema4).

**References:**

1. Li H., Durbin R. Fast and accurate short read alignment with Burrows–Wheeler transform. *Bioinformatics* 2009;**25**(14):1754–60. Doi: 10.1093/bioinformatics/btp324.

2. McKenna A., Hanna M., Banks E., Sivachenko A., Cibulskis K., Kernytsky A., et al. The Genome Analysis Toolkit: A MapReduce framework for analyzing next-generation DNA sequencing data. *Genome Res* 2010;**20**(9):1297–303. Doi: 10.1101/gr.107524.110.

3. Danecek P., Bonfield JK., Liddle J., Marshall J., Ohan V., Pollard MO., et al. Twelve years of SAMtools and BCFtools. *GigaScience* 2021;**10**(2):giab008. Doi: 10.1093/gigascience/giab008.

4. Manichaikul A., Mychaleckyj JC., Rich SS., Daly K., Sale M., Chen W-M. Robust relationship inference in genome-wide association studies. *Bioinformatics* 2010;**26**(22):2867–73. Doi: 10.1093/bioinformatics/btq559.

5. Verma A., Damrauer SM., Naseer N., Weaver J., Kripke CM., Guare L., et al. The Penn Medicine BioBank: Towards a Genomics-Enabled Learning Healthcare System to Accelerate Precision Medicine in a Diverse Population. *J Pers Med* 2022;**12**(12):1974. Doi: 10.3390/jpm12121974.

6. Szustakowski JD., Balasubramanian S., Kvikstad E., Khalid S., Bronson PG., Sasson A., et al. Advancing human genetics research and drug discovery through exome sequencing of the UK Biobank. *Nat Genet* 2021;**53**(7):942–8. Doi: 10.1038/s41588-021-00885-0.

7. Taliun D., Harris DN., Kessler MD., Carlson J., Szpiech ZA., Torres R., et al. Sequencing of 53,831 diverse genomes from the NHLBI TOPMed Program. *Nature* 2021;**590**(7845):290–9. Doi: 10.1038/s41586-021-03205-y.

8. Auton A., Abecasis GR., Altshuler DM., Durbin RM., Abecasis GR., Bentley DR., et al. A global reference for human genetic variation. *Nature* 2015;**526**(7571):68–74. Doi: 10.1038/nature15393.

9. Alexander DH., Novembre J., Lange K. Fast model-based estimation of ancestry in unrelated individuals. *Genome Res* 2009;**19**(9):1655–64. Doi: 10.1101/gr.094052.109.

10. Jin Y., Schaffer AA., Feolo M., Holmes JB., Kattman BL. GRAF-pop: A Fast Distance-Based Method To Infer Subject Ancestry from Multiple Genotype Datasets Without Principal Components Analysis. *G3 (Bethesda)* 2019;**9**(8):2447–61. Doi: 10.1534/g3.118.200925.

11. Purcell S., Neale B., Todd-Brown K., Thomas L., Ferreira MAR., Bender D., et al. PLINK: a tool set for whole-genome association and population-based linkage analyses. *Am J Hum Genet* 2007;**81**(3):559–75. Doi: 10.1086/519795.
