# Supplementary figures and images for: Phenome-wide association study of monogenic inflammatory bowel disease genes in diverse biobanks identifies population-specific and shared Goldilocks alleles: implications for Precision Medicine
Source: J Crohns Colitis. 2025 Aug 5;19(7):jjaf098. doi: 10.1093/ecco-jcc/jjaf098 (PMC13223577; doi:10.1093/ecco-jcc/jjaf098)

A

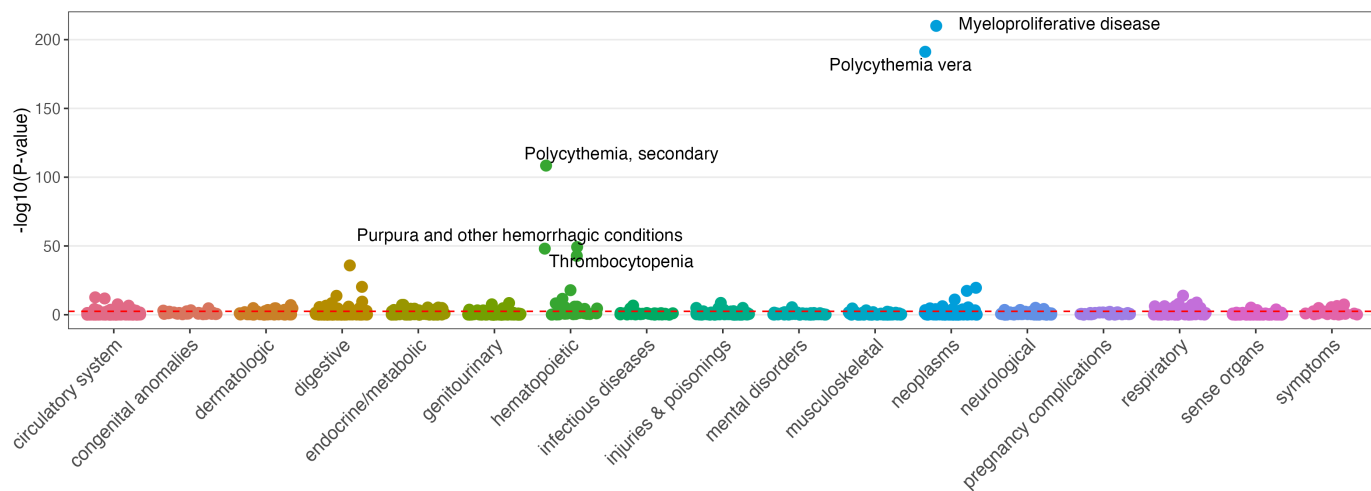

B

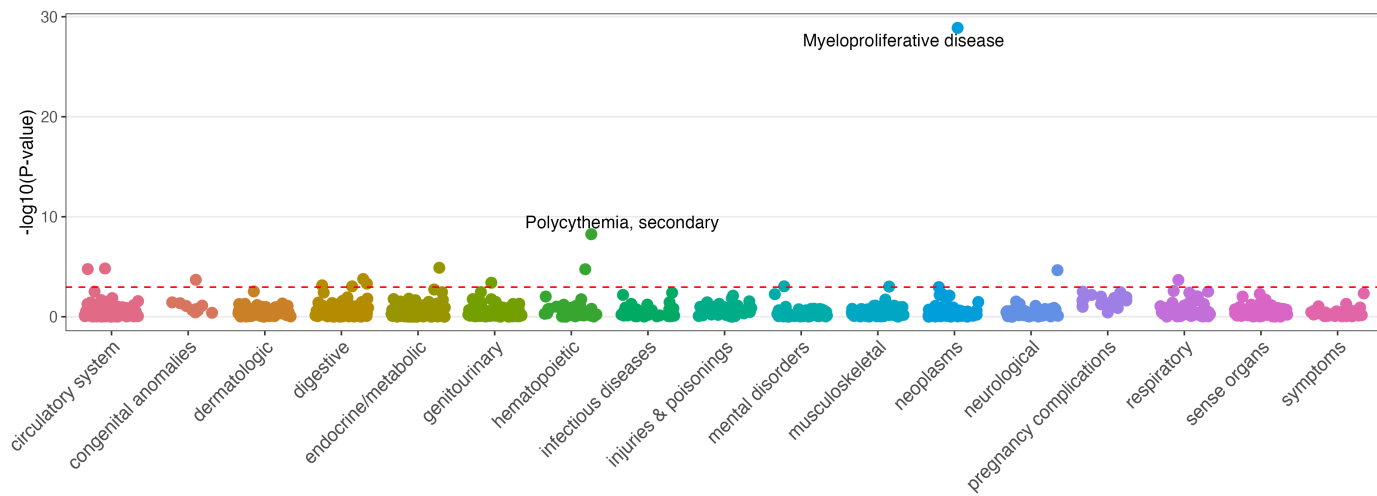

Supplement: jjaf098_suppl_Supplementary_Figures_1-2_Tables_1-10 [file jjaf098_suppl_supplementary_figures_1-2_tables_1-10.zip › jjaf098_Suppl_Methods_Tables 1-10_Figures 1-2/Suppfigure2.pdf]
